# FEASIBILITY OF FALLS PREVENTION PROGRAMS IMPLEMENTATION BASED ON ACADEMIC-COMMUNITY PARTNERSHIPS

**DOI:** 10.1093/geroni/igac059.3097

**Published:** 2022-12-20

**Authors:** Uma Kelekar, Cathy Elrod, Patricia Heyn, Rita Wong

**Affiliations:** Marymount University, Arlington, Virginia, United States; Marymount University, Arlington, Virginia, United States; Center for Optimal Aging, Marymount University, Fairfax Station, Virginia, United States; Marymount University, Arlington, Virginia, United States

## Abstract

The Northern Virginia (NoVa) region is diverse in age, race, ethnicity, economic status, health status, culture, and language. Based on state data, it is estimated that 28.7% of the 75,000 older adults in NoVa will experience at least one fall annually. Since 2014, the Administration for Community Living (ACL) has sponsored implementation of evidence-based fall prevention programs (EBFPPs). The aims of this study are to 1) describe the EBFPP participants and delivery sites and 2) report initial effectiveness. Marymount University (MU) received two cooperative agreement grants (2016, 2018) from the ACL to implement in NoVa three EBFPPs: 1) Stay Active and Independent for Life (SAIL), 2) A Matter of Balance (MOB), and 3) Otago Exercise Program (OEP). The Northern Virginia Falls Prevention Alliance was formed and a Regional Training Office was built. The data source is the ACL national falls database. From 2016–2022, over 5,000 older adults were reached, over 400 EBFPP leaders were trained, and programs were delivered at 60 sites. Data analysis indicates that falls decreased from 20% to 15%. Those participating in MOB showed the greatest decline in falls (from 34% to 14%). Fall injuries declined from 11% to 7% and ED visits decreased from 3% to 1%. Preliminary analysis indicates beneficial impact: this academic-community partnership has reached people at low risk of falling (SAIL), and had an impactful decline in falls for individuals at moderate to high risk (MOB). Future research will investigate program outcome differences based on other demographics and delivery models.

